# Beyond the Paclitaxel and Vinca Alkaloids: Next Generation of Plant-Derived Microtubule-Targeting Agents with Potential Anticancer Activity

**DOI:** 10.3390/cancers12071721

**Published:** 2020-06-29

**Authors:** Dangquan Zhang, Arun Kanakkanthara

**Affiliations:** 1 College of Forestry, Henan Agricultural University, Zhengzhou 450002, China; 2 Division of Oncology Research, Mayo Clinic, Rochester, MN 55905, USA; 3 Department of Molecular Pharmacology and Experimental Therapeutics, Mayo Clinic, Rochester, MN 55905, USA

**Keywords:** microtubule-targeting agents, microtubule stabilizing agents, microtubule destabilizing agents, tubulin binding site

## Abstract

Plants are an important source of chemically diverse natural products that target microtubules, one of the most successful targets in cancer therapy. Colchicine, paclitaxel, and vinca alkaloids are the earliest plant-derived microtubule-targeting agents (MTAs), and paclitaxel and vinca alkaloids are currently important drugs used in the treatment of cancer. Several additional plant-derived compounds that act on microtubules with improved anticancer activity are at varying stages of development. Here, we move beyond the well-discussed paclitaxel and vinca alkaloids to present other promising plant-derived MTAs with potential for development as anticancer agents. Various biological and biochemical aspects are discussed. We hope that the review will provide guidance for further exploration and identification of more effective, novel MTAs derived from plant sources.

## 1. Introduction

Microtubules are the major components of the eukaryotic cytoskeleton. They are composed of α- and β-tubulin heterodimers that interconvert between phases of rapid growth (polymerization) and shrinkage (depolymerization) [1]. Microtubules are central to several important cellular activities, including maintenance of cell shape and cell motility, accurate chromosome segregation during mitosis, and intracellular trafficking of macromolecules and organelles in the interphase [2,3,4].

**Figure 1:**
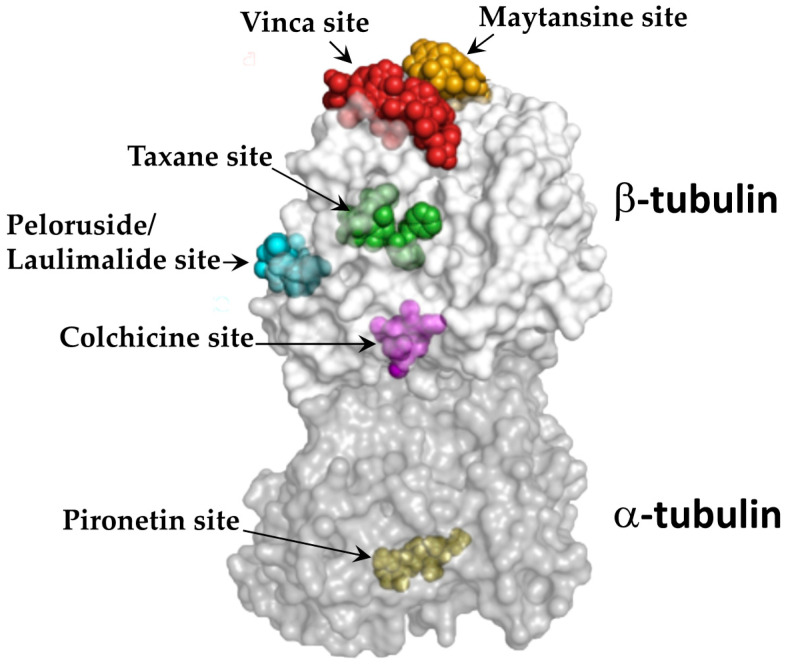
The known binding sites of microtubule-targeting agents on tubulin. The α-tubulin (dark grey) and β-tubulin (light grey) dimers with bound ligands are presented in semitransparent surface. The representative ligand structures for each site were superimposed onto their appropriate binding sites. The image is adapted from Steinmetz MO and Prota AE [14] by obtaining Copyright clearance from the publisher, Elsevier, and the permission from the authors.

The crucial roles played by microtubules in both mitotic and interphase cellular functions make them important anticancer drug targets. Accordingly, microtubule-targeting agents (MTAs) inhibit the proliferation of cancer cells by disrupting interphase cell signaling events and/or preventing the precise functioning of spindle microtubules, both of which ultimately induce cell death via apoptosis [4]. Notably, additional mechanisms may also contribute to the effects of MTAs against cancer cells, such as the interplay of MTAs with secondary targets, including microtubule-associated proteins and other signal transductors [5]. MTAs are, however, broadly classified into two categories: microtubule stabilizing agents and microtubule destabilizing agents. Microtubule stabilizing agents are a class of drugs that promote tubulin polymerization and stabilize microtubules against depolymerization [6]. In contrast, microtubule destabilizing agents depolymerize existing microtubules and/or prevent tubulin heterodimers from forming polymers [6]. Microtubule stabilizing agents are further classified into two types based on their tubulin binding sites: taxane-site binding agents (e.g., paclitaxel, docetaxel, discodermolide, epothilones, and zampanolide) [7,8] and peloruside/laulimalide-site binding agents (e.g., peloruside A and laulimalide) [9] (Figure 1). Microtubule destabilizing agents are classified into the vinca domain-binding agents (e.g., vinblastine, vincristine, and halichondrin B) [10], the colchicine domain-binding agents (e.g., combretastatins and 2-methoxyestradiol) [11], the maytansine site-binding agents (e.g., maytansine, rhizoxin, and PM60184) [12], and the pironetin site-binding agents (e.g., pironetin) [13] (Figure 1). Several new MTAs that occupy these sites and exert remarkable anticancer activities have been discovered, with plants being one of the major sources.

The best examples of plant-derived MTAs are paclitaxel (Taxol ^®^) and vinca alkaloids, which are important treatments for many different cancers, such as ovarian, breast, bladder, prostate, and lung cancers, and lymphoma [7,15]. Although usually used to treat gout, colchicine, the first compound identified as an MTA, was also isolated from a plant [16]. Intriguingly, recent estimates indicate that the plant kingdom comprises at least 500,000 species and only less than 10 percent of those have been phytochemically investigated for pharmacological applications [17], suggesting that many new compounds that may target microtubules remain to be discovered.

Understanding the biological and biochemical features of existing plant-derived MTAs is important for identifying novel, more effective antimicrotubule agents. The advances in paclitaxel and vinca alkaloids have been discussed multiple times in detail lately [11,18,19,20,21,22]. Accordingly, this review is focused on other plant-derived MTAs that have the potential for development as anticancer agents. Only compounds (1) that exhibited bioactivity (termed to cytotoxic activity against cancer cells in culture and/or in xenografts here onwards) superior to paclitaxel and vinca alkaloids, or (2) that underwent clinical trials are discussed.

## 2. Microtubule Stabilizing Agents

### 2.1. Taccalonolides

**Figure 2:**
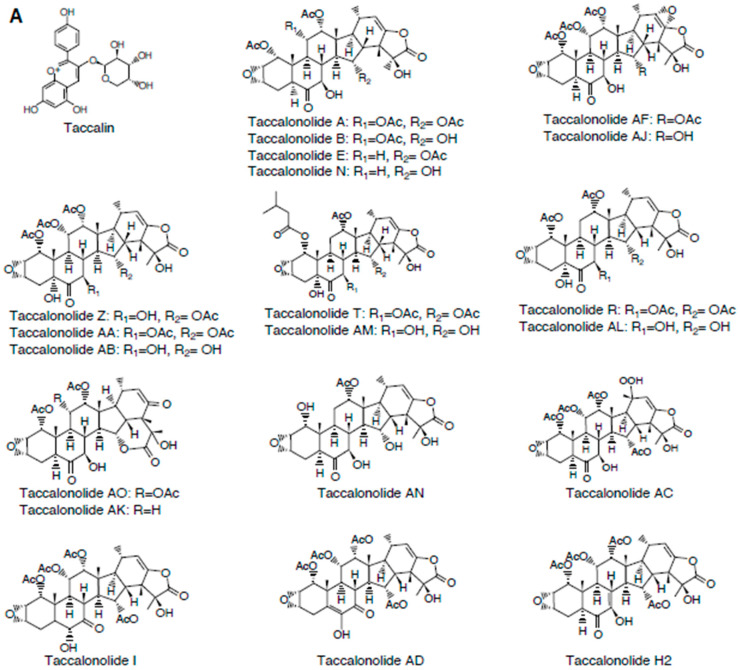

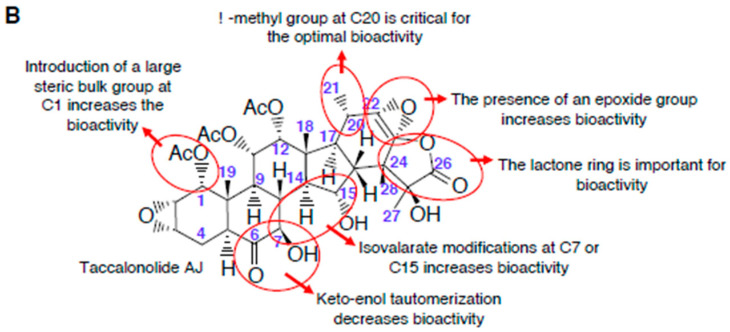
(**A**) Chemical structures of taccalonolides. (**B**) Structure-activity relationships of taccalonolides.

Taccalonolides are the best-studied plant-derived microtubule stabilizing agents after the taxanes. Taccalonolides were isolated from the plants of the genus *Tacca*, and about 38 taccalonolides (A-Z, AA-AJ, AK-AN, and H2) had been obtained from various *Tacca sp.* or through semi-synthesis [23] (Figure 2). Taccalin was the first compound isolated from the *Tacca* plants (Figure 2). In 2003, a cell-based study with taccalonolides A and E by Mooberry’s group provided the first evidence that taccalonolides have a microtubule stabilizing property [24].

#### 2.1.1. Mechanism of Action

Taccalonolides have a unique structure, with some of them exhibiting a distinct microtubule stabilizing property as compared to other microtubule stabilizing agents. For example, unlike paclitaxel, the earliest taccalonolides, A and E, failed to induce assembly of purified tubulin in vitro [24]. However, both the compounds caused paclitaxel-like effects on microtubules inside cells, including induction of microtubule bundling in interphase cells and multiple asters in mitotic cells [24], suggesting that these taccalonolides possess a microtubule stabilizing mechanism that is independent of a direct interaction with microtubules. How exactly taccalonolides A/E stabilize microtubules, without directly interacting with microtubules in cells is not clear, but one potential explanation might be that the taccalonolides are prodrugs that are, in cells, modified into taccalonolides that are capable of binding to microtubules.

Identified later, the more potent taccalonolides AF and AJ (Figure 2) showed for the first time a direct interaction with microtubules [25,26].

#### 2.1.2. Tubulin Binding Sites

Extensive studies showed that taccalanolides AF and AJ covalently bind to the taxane-site on β-tubulin [25,27]. Notably, to date, only three other microtubule stabilizing agents, zampanolide, dactylolide, and cyclostreptin, have been reported to react covalently with tubulin [28,29]. Taccalonolide AJ covalently interacted with tubulin in a similar manner to cyclostreptin [27]. The 2.05 Å crystal structure demonstrated that taccalonolide AJ covalently bind to β-tubulin residue D226 using its C22–C23 epoxide group [25]. The AJ binding induced a closed-to-open and a loop-to-helix conformational shift of β-tubulin M-loop, both of which have been proposed to facilitate lateral tubulin interactions and microtubule assembly [25]. Additionally, taccalonolide AJ binding locked the β-tubulin E-site into a GTP-binding-competent conformation that inhibit GTP hydrolysis [25].

#### 2.1.3. Structure-Activity Relationships

Comprehensive structure-activity relationships of taccalonolides have been described, owing to the availability of a series of structurally diverse natural and semi-synthetic taccalonolides (Figure 2A,B). Studies with taccalonolide analogues, AO and AK, that have structural rearrangements at C20-C23 revealed that E-ring constituents at C20-C23 of taccalanolide backbone play an important role in promoting their microtubule stabilizing and bioactivity [30] (Table 1A). Likewise, epoxidation of the C22-C23 double bond had a positive effect on taccalonolide bioactivity [31]. This is evident from the improved bioactivity of taccalonolides AF and AJ relative to their parent compounds taccalonolides A and B, respectively [26,31] (Table 1A). The presence of a large, steric bulk group at C1 also increases the bioactivity of taccalonolides. This was first recognized from a ~39-fold increase in the antiproliferative effect of taccalonolide T compared to taccalonolide R [32] (Table 1A). Taccalonolide R contains an acetoxy group at C1, while T contains an isovalerate group. Consistently, a 17-fold increase in bioactivity was observed when the acetoxy group at C1 in taccalonolide AL was replaced with an isovalerate group in taccalonolide AM [30].

A reduced bioactivity was found for taccalonolide AC that contains an α-hydroperoxyl group at C20 [30]. Most taccalonolides contain an α-methyl group at C20, suggesting that the α-methyl group at C20 is critical for the optimal bioactivity of taccalonolides (Table 1A).

Modification at C5 of the taccalonolide molecule also affects its efficacy. The C5-hydroxy group is the only structural difference between the taccalonolides N and AL, but this change was enough to contribute to a 4-fold decrease in efficacy for taccalonolide AL [30] (Table 1A). On the contrary, the presence of C5 hydroxy group in taccalonolide AZ resulted in a 44-fold increase in bioactivity compared to taccalonolide A, which lacks the C5 hydroxy group [23,32]. Additionally, although taccalonolides B and AB have a C5 hydroxy group, it did not markedly affect their efficacy [32]. As such, the significance of C5 hydroxy group on the bioactivity of taccalonolides seems to be complex, but one possibility could be that the modifications at C5 alone may not have any effect on bioactivity, but it may have an effect when it is combined with other modifications.

The C5–8 region in the B-ring of the taccalonolide molecule also mediates the effects of the compound (Figure 2; Table 1A). This is evidenced by the 15-fold decrease in the bioactivity of taccalonolide I compared to taccalonolide B, where taccalonolide I was derived by a keto-enol tautomerization between the C6 ketone and C7 hydroxy groups on taccalonolide B [30]. Notably, when this arrangement together with a double bond at C5-C6 was present, the bioactivity was increased, which is clear from the difference in potencies of taccalonolides AD and A [23,30]. Moreover, the presence of a C7-C8 double bond increased the potency to 7-fold. These suggest that the B ring of the taccalonolide backbone with a ketone at C6 position and double bonds at C5-C6 and/or C7-C8 may be critical for the efficacy of taccalonolides [23,30].

The substituents at C7 and C15 of the taccalonolide also determines the efficacy of the agent [33] (Table 1A). Taccalonolide AF, which contains a C15 acetoxy group, exhibited superior bioactivity in in vivo tumor xenograft models compared to taccalonolide AJ that contain a C15 hydroxy group [33,34] (Table 1A,B). A thorough analysis of bioactivities of 28 new semisynthetic taccalonolide analogues with various monosubstitutions at C-7 or C-15 or disubstitutions at C-7 and C-25 demonstrated that isovalerate modifications at C7 or C15 increase potency and antitumor activity in a drug-resistant xenograft model [33]. Collectively, these comprehensive structure–activity relationship studies pinpoint the key determinants of taccalonolide potency, and provide important insights into rational design of new anticancer leads based on this class of agents.

**Table 1:** (**A**) Half-maximal inhibitory concentrations (IC50) of taccalonolides in HeLa cells. (**B**) Details of in vivo tumor xenograft studies in mice using taccalonolides AF and AJ. IC50 of (**C**) persin and its analogues, and (**D**) curcumin, maytansine, combretastatin, noscapine, and quercetin in various cancer cell lines.

(**A**)																																													
**Compound**			**IC_50_ (μM)**				**Compound**					**IC_50_ (μM)**					**Compound**				**IC_50_ (μM)**				**Compound**				**IC_50_ (μM)**				**Compound**				**IC_50_**		**Compound**				**IC_50_ (μM)**		References: [24,26,30,31,32]
Taccalonolide A			5.32 ± 0.23				Taccalonolide N					8.5 ± 0.40					Taccalonolide Z				0.12 ± 0.008				Taccalonolide AD				3.4 ± 0.2				Taccalonolide AO				>50		Taccalonolide AN				1.5 ± 0.1		
Taccalonolide B			3.12 ± 0.18				Taccalonolide I					49.2 ± 2.8					Taccalonolide AA				0.032 ± 0.002				Taccalonolide AE				5.0 ± 0.2				Taccalonolide AK				>50		Paclitaxel				0.0012 ± 0.1		
Taccalonolide E			39.5 ± 4.70				Taccalonolide R					13.0 ± 1.0					Taccalonolide AB				2.7 ± 0.1				Taccalonolide AF				0.023 ± 0.003				Taccalonolide AL				34.4 ± 7.5								
Taccalonolide H2			0.73 ± 0.02				Taccalonolide T					0.34 ± 0.02					Taccalonolide AC				>50				Taccalonolide AJ				0.0042 ± 0.0003				Taccalonolide AM				2.0 ± 0.1						
(**B**)																																													
**Compound**									**Xenograft Models**									**Method of Tumor Cell Administration**													**Treatment Strategy/Dose**														**References**
Taccalonolide AF and AJ									MDA-MB-231 breast cancer									intraperitoneal													1. Taccalonolide AF: 2 mg/kg on Days 1, 4, 8 2. Taccalonolide AF: 2.5 mg/kg on Days 1 and 5 3. Taccalonolide AJ: 0.5 mg/kg on Days 1, 3, 5, and 8														[27]
Taccalonolide AF and AJ									SCC-4 oral cancer cells									subcutaneous													1. Taccalonolide AF: 80 μg on Days 0 and 3 2. Taccalonolide AJ: 40 μg on Days 0 and 3 3. Taccalonolide AJ: 80 μg on Days 0 and 3														[34]
(**C**)																																													
	**Breast Cancer Cell Lines**																									**Ovarian Cancer Cell Lines**												**Prostate Cancer Cell Lines**						**Leukemia Cell Lines**	References: [35,36,37]
Compound (μM)	MCF-7			T-47D		MDA-MB-468					MDA-MB-157			SK-BR3		Hs578T				MDA-MB-231				MCF-10A		OVCAR-3				IGROV-1				1A9		A2780		PC-3			LNCaP			HL-60	
Persin	15.1 ± 1.3			30.3 ± 2.3		25.0 ± 2.8					12.8 ± 1.2			19.7 ± 1.3		32.1 ± 2.3				>39				>39		27.9 ± 4.5				15.6 ± 3.6				13.7 ± 0.6		8.1 ± 1.1		30.0 ± 3.0			22.0 ± 1.8			1.9 ± 0.1	
1	17.1 ± 1.7			20.7 ± 3.2		>39					>39			>39		>39				>39				>39		>39				>39				4.1 ± 0.4		8.1 ± 1.4		>39			>39			0.6 ± 0.03	
2	>32																																	18.9 ± 1.3		13.7 ± 0.9								4.0 ± 0.1	
3	27.7 ± 5.5																																	19.4 ± 2.2										2.6 ± 0.4	
4	>27																																	21.2 ± 1.8										7.5 ± 0.2	
5	23.8 ± 2.2																																	34.1 ± 5.3										5.8 ± 0.1	
6	29.0 ± 4.2																																	47.6 ± 3.5										28.4 ± 0.5	
7	>21																																												
8	>24																																												
9	20.1 ± 3.6																																												
10	>65																																	124 ± 20										22.8 ± 1.0	
(**D**)																																													
		**Breast Cancer Cell Lines**													**Lung Cancer Cell Lines**												**Squamous Carcinoma Cell Lines**					**Lymphoma Cell Lines**			**Ovarian Cancer Cell Line**			**Cervical Cancer Cell Line**		**Leukemia Cell Line**		**Prostate Cancer Cell Line**			**References**
Compound		MCF7			MDA-MB-231			BT-474		SK-BR3			MDA-MB-435		A594				H1299			H292	NCI-H358M				Tu212	Tu686				BJAB			OVCAR-8			HeLa		HL60		LNCap		PC3M	
Curcumin (μM)															11.2				6.03			11.6					5.5	6.4										25.0							[38,39,40,41,42]
Maytansine (pM)		30						420		44																						270													[43,44,45]
Combretastatin A4 (nM)					2.8								5.3		3.8								8												0.37			0.9		2.1				4.7	[46,47]
Noscapine (μM)		29			69																																								[48]
Quercetin (μM)		14													1																											22			[49]

#### 2.1.4. Advantages over Paclitaxel

Mutations in the taxoid site on β-tubulin and overexpression of the Pgp drug efflux pump or βIII-tubulin are common mechanisms of resistance to the taxanes and vinca alkaloids [6]. Notably, taccalonolides were able to overcome these resistance mechanisms by cancer cells [50]. Moreover, taccalonolides exhibited excellent in vivo antitumor activity in Pgp-overexpressing, paclitaxel-/doxorubicin-resistant mouse tumor models [27,34,50]. This improved efficacy of taccalonolides may be explained by its high degree of cellular retention compared to paclitaxel that could be potentially stemmed from their covalent interaction with tubulin [51]. Together, taccalanolides represent an unique class of microtubule stabilizing agents with anticancer properties that are potentially superior to paclitaxel.

### 2.2. Persin

**Figure 3:**
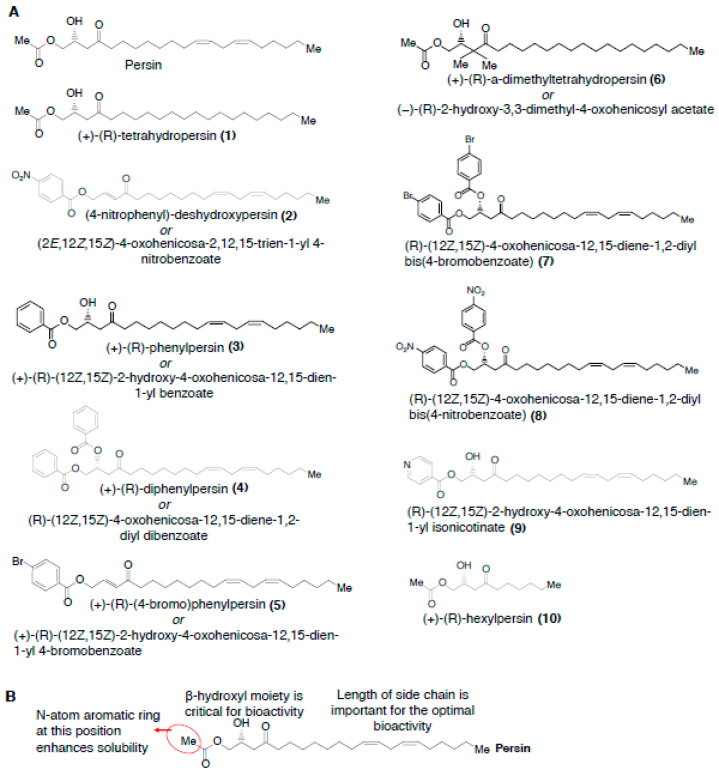
(**A**) Chemical structures of persin and persin analogues. (**B**) Structure-activity relationships of persin.

Persin ((+)-(R)-persin) is a polyketide long-chain lipid with strong structural homology to linoleic acid, and is synthesized in idioblast oil cells present in avocado leaves and fruit [52]. Persin contains a β-hydroxy ketone system, which is flanked on one side by an acetate group and on the other side by a long, partially unsaturated hydrocarbon chain (Figure 3).

#### 2.2.1. Mechanism of Action

Although persin was first isolated in 1975 from the leaves of the avocado plant, *Persea americana* Mill. (Lauraceae) [53], its microtubule stabilizing property, was identified only in 2006 [35]. In the subsequent years, a number of studies have confirmed the antiproliferative effects of persin against various cancer cell types [35,36,37,54]. Notably, persin increased tubulin polymerization, caused G_2_/M arrest, and also synergized with other microtubule stabilizing agents in ovarian cancer cells [36]. Moreover, in lactating Quackenbush mice, dosing of persin caused severe necrosis and/or apoptosis of the mammary gland, with no visible effects on other tissues [55]. Other MTAs, such as vinca alkaloids and colchicine, have also affected the lactating mammary gland by an unknown mechanism (reviewed in a previous study [56]). Thus, given that vinca alkaloids and colchicine are currently in clinical use for treating cancer and gout, respectively, it seems less likely that the effect of persin on mammary gland may limit its potential as a clinical lead compound.

#### 2.2.2. Tubulin Binding Sites

The exact binding site of persin on tubulin is not known. However, a cell-based Flutax-1 (a fluorescent taxol derivative) competitive binding assay showed that persin displaces Flutax-1 from tubulin, suggesting that persin may occupy a site adjacent to or overlapping with the taxoid-site on β-tubulin [36]. Although this is apparently contradictory to the observations that taxoid site mutations had no effect on persin bioactivity and that persin synergized with paclitaxel [36], similar results have been seen with other taxoid site-binding microtubule stabilizing agents before. For example, microtubule stabilizing agents, discodermolide and zampanolide, that are known to bind in the taxoid site have been active in taxoid-site-mutated paclitaxel-resistant cells and, in addition, they have been shown to synergize with paclitaxel [57,58]. Because the existing studies does not provide direct evidence for a persin-tubulin interaction [35,36], more binding studies with purified tubulin coupled with other approaches, such as hydrogen/deuterium exchange mass spectrometry and computational modeling, are warranted to understand the mode of persin-tubulin interaction.

#### 2.2.3. Structure-Activity Relationships

Several analogues of the persin have been synthesized (Figure 3A,B), and analysis of their bioactivities have revealed structural features important for their antiproliferative effects [36,37]. Upon comparison of the persin analogs, **1** and **10**, reduction in the length of side chain is the only structural difference (Figure 3A,B). In bioactivity studies using breast cancer cells, **1** exhibited significant antiproliferative effects (with an IC_50_ of ~17 μM); whilst **10** was devoid of any antiproliferative effects even at concentration as high as 65 μM [37] (Table 1C), indicating that the length of side chain, but not the presence of unsaturation, is optimal for persin’s bioactivity. However, intriguingly, the short-chain α, β- unsaturated β’-hydroxy-, -acetoxy, and -phenoxy-ketone analogs were active against breast, colon, ovarian, non small-cell lung cancers, and malignant pleural mesothelioma cell lines [59,60,61]. In addition, the short-chain analogs also potentiated the effects of paclitaxel in pancreatic ductal adenocarcinoma cells [59,61]. It could thus be possible that, compared to **10**, these compounds bind differently to its tubulin target, which, in turn, might be contributing to their better bioactivity vs. that of **10**.

Comparing the activity of compounds **3** and **5** suggests that there is a lipophilic bulk-tolerance at the far left end of the molecule [37] (Figure 3A,B). In addition, a slightly greater bioactivity of **5** compared to **3** may suggest that an electronic-deficient aromatic system is favored in the position [37] (Table 1). The observation that bis-aroylated compounds, **4**, **7**, and **8**, and the enone, **2**, were inactive in cells indicates that the β-hydroxyl moiety is important for persin’s bioactivity [37]. Notably, the pyridinyl compound, **9**, exhibited a similar lipophilicity as persin, and showed comparable activity to the persin [37] (Figure 3, Table 1C). The solubility of the **9** is seemingly enhanced by the greater polarity imparted by the N-atom aromatic ring of the molecule, suggesting that further exploration of the heteroaryl analogs of persin may be useful.

#### 2.2.4. Persin Activity in MTA-Resistant Cells

Growth inhibitory assays with persin and two of its analogues, **1** and **2**, in Pgp overexpressing multidrug-resistant ovarian cancer cells revealed that the compounds are not substrates for the drug efflux pump [36]. Additionally, the persins were active in ovarian cancer cells that are resistant to taxanes, epothilone, and peloruside due to acquired mutations in their β-tubulin binding sites [36]. The ability of persin to bypass the common clinically-relevant mechanisms of resistance to MTAs suggests that further studies on persin could prove fruitful.

## 3. Microtubule Destabilizing Agents

### 3.1. Curcumin

**Figure 4:**
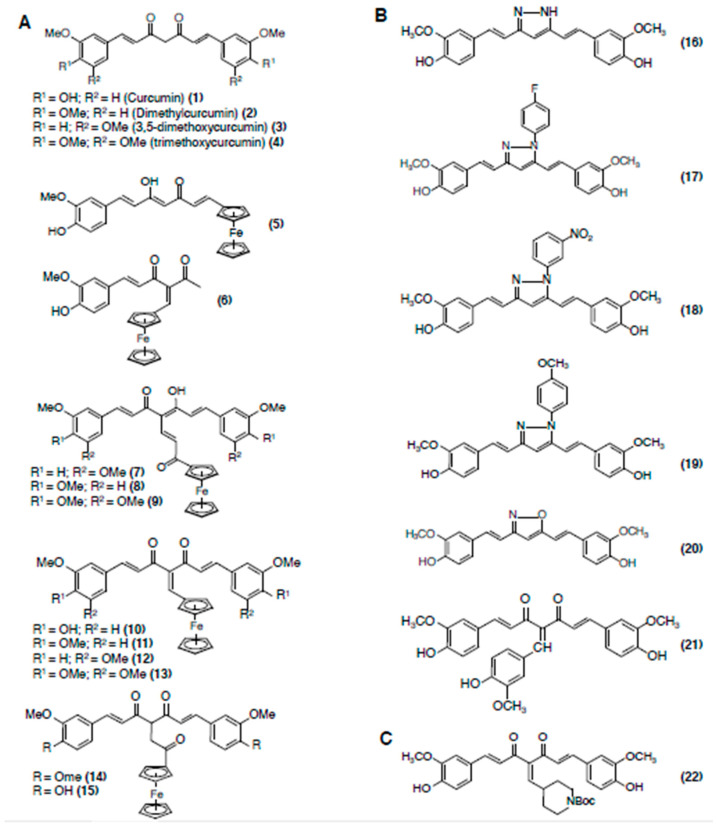
Chemical structures of curcumin and curcumin analogs. (**A**) Ferrocenyl curcumin derivatives. (**B**) Pyrazole, isoxazole, and benzylidiene derivatives of curcumin. (**C**) A highly potent curcumin derivative, named C1 (**22**).

Curcumin is a polyphenolic compound originally isolated from the rhizome of turmeric, *Curcuma longa* [62] (Figure 4). Numerous cell culture and animal studies have demonstrated remarkable antiproliferative effects for curcumin against diverse cancer types [38,39] (Table 1D). Moreover, curcumin has already completed many cancer clinical trials, and there are several ongoing trials exploring the efficacy of curcumin as single-agent and in combination with other chemotherapeutic agents against various cancers [63].

#### 3.1.1. Mechanism of Action

Curcumin directly binds to tubulin, reduces GTPase activity, and inhibits tubulin polymerization [40,41,42]. Because curcumin exerts a multitude of biological effects potentially through regulation of various molecular targets, it is not clear if disruption of microtubules is the sole mechanism underlying its anticancer properties [40,41,42].

#### 3.1.2. Tubulin Binding Sites

Initial studies using purified tubulin showed that colchicine-site binding agents modestly prevent curcumin-tubulin interaction, indicating that curcumin and colchicine may share an overlapping site on tubulin [64]. However, a more detailed study by Chakraborti et al. [41] later demonstrated that the tubulin binding site of curcumin is located at the interdimer interface that is about 32 Å away from the colchicine-site. The curcumin binding site involves β-tubulin residues 96–98 and α-tubulin residues 251–256 [41]. Moreover, the binding pocket contained H3′-helix (residues 105–110), T4 and T5 loops (residues 130–133 and 163–165), and the H11′ helix (residues 407–411) [41], thus demonstrating that curcumin occupies a unique site that partly overlap with the colchicine-site on tubulin.

#### 3.1.3. Structure-Activity Relationships

Several curcumin ferrocenyl derivatives have been synthesized [65,66]. The curcumin ferrocenyl derivatives were synthesized by covalent anchorage of three different ferrocenyl ligands to organic curcuminoids substituted with methoxyl and hydroxyl groups on the aromatic rings. In in vitro tubulin polymerization inhibition assays, the ferrocenyl propenone curcuminoids (compounds **7**, **8**, **9**), ferrocenyl methylene curcuminoids (compounds **10**, **11**, **12**, **13**), and the ferrocenyl ethanone curcuminoids (compounds **14** and **15**), showed much better tubulin polymerization inhibition acitivity than curcumin (Figure 4A–C).

Chakraborti et al. also synthesized several curcumin analogues [41] (Figure 4). They include: (1) pyrazole derivatives of curcumin (compounds **16**–**19**), (2) an isoxazole derivative of curcumin (compound **20**), and (3) a benzylidiene derivative of curcumin (compound **21**) [41] (Figure 4). The pyrazole (compounds **16**–**19**) and isoxazole (compound **20**) curcumins docked in to the curcumin binding site on tubulin and showed a greater increase in stability than curcumin at physiological pH and reducing atmosphere [67]. The benzylidiene derivative (compound **21**) bound tubulin with a higher affinity compared to curcumin, and it was associated with increased ability to prevent microtubule assembly and induce cancer cell death. The **21** has free diketone moieties and a substituted polyphenol ring in between the dicarbonyl moiety, suggesting that the extra steric hindrance caused by the substitution of a polyphenol in between the diketones may make the compound conformationally more favorable to bind with tubulin. Alternatively, substitution of a polyphenol ring in between the diketones may introduce a tridentate molecule that can bind tubulin with higher affinity.

Curcumin-derived compound C1 (compound **22**) is one of most active curcumin derivatives [68] (Figure 4C). Compound **22** inhibited the microtubule assembly, perturbed the lattice structure of microtubules, suppressed their GTPase activity, and inhibited cancer cell proliferation much more effectively than curcumin [68]. Importantly, **22** also showed more stability in aqueous buffer than curcumin [68], suggesting that the enhanced biological activity of **22** may be partly due to its increased stability in solution.

More recently, curcumin mimics bearing an additional bridged phenyl ring in conjugation [69], and a series of curcumin inspired imidazo [1,2-a]pyridine analogues [70] and indole analogues [71] have been synthesized. Several of these analogues efficiently blocked tubulin polymerization and exerted improved antiproliferative effects against various cancer cell lines.

### 3.2. Combretastatins

**Figure 5:**
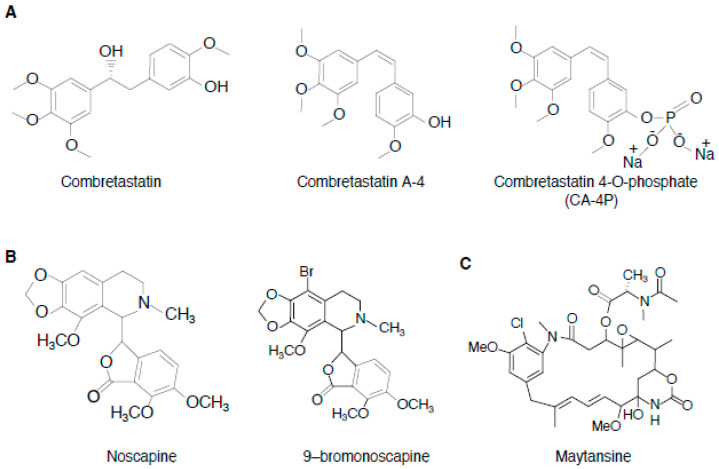
Chemical structures of (**A**) combretastatins, (**B**) noscapine and 9-bromonoscapine, and (**C**) maytansine.

Combretastatins are a class of natural stilbene that were originally isolated from the bark of African willow tree *Combretum caffrum* [72]. Combretastatins A1 and B1, isolated in the late 1980s, are the first-known combretastatins with microtubule-targeting activity [72]. Since then, several related molecules and combretastatin derivatives have been synthesized, including compounds modified on the double bond with various heterocyclic rings such as isoxazole, indole, β-lactam, trans-methylpyrazoline, pyrazole, pyrazoline, cyclohexenone, and oxadiazoline [46,73,74]. Of those, combretastatin A-4 (CA-4) has shown to be among the most effective [47] (Figure 5A, Table 1D). CA-4 also exhibited a lower toxicity profile than paclitaxel and the vinca alkaloids [46]. Although its poor water solubility hampered its clinical applicability, several water-soluble CA-4 prodrugs have been generated over the years, the most studied of which is its 4-O-phosphate (CA-4P) [75] (Figure 5A). CA-4P is rapidly converted into active CA-4 by nonspecific endogenous phosphatases present in plasma and on endothelial cells.

CA-4P has completed several cancer clinical trials as monotherapy and in combination with other treatments, including antiangiogenic therapy and chemotherapy [46,76]. In a Phase I clinical trial [77], CA-4P was delivered to 34 patients by a 10-min weekly infusion for 3 weeks followed by a week gap, with intrapatient dose escalation. The starting dose was 5 mg/m^2^ and the dose escalation was achieved by doubling until grade 2 toxicity was seen. Notably, CA4P was generally well tolerated, with only mild nausea or vomiting that was easily controlled by antiemetics, and the dose-limiting toxicity was reversible ataxia. The cardiovascular adverse events were only restricted to changes in pulse and blood pressure, with no cardiac events. Moreover, in contrast to conventional chemotherapy, CA-4P showed a different set of toxicity profile with no neutropenia or thrombocytopenia, but a mild lymphocytopenia at higher doses. Together, these findings highlight that CA-4 is an important clinical lead compound for cancer therapy.

#### 3.2.1. Mechanism of Action and the Tubulin Binding Sites

Combrestatins have a structural similarity to colchicine and bind to tubulin at the same domain, preventing microtubule polymerization [78]. Combrestatins acts primarily on the vascular endothelial cells of the tumor, which causes tumor vasoconstriction that leads to cancer cell death, due to insufficient blood supply [79].

#### 3.2.2. Structure–Activity Relationships

Because of its simple structure, hundreds of CA-4 analogs have been synthesized, with some having activity against paclitaxel-resistant cancer cells and some having different tubulin binding mechanism from that of CA-4 (e.g., cyclopropylamide analogs) [80]. Structure–activity studies demonstrated that the double bond in the cis configuration of its stilbene moiety and the presence of 3,4,5-trimethoxy-substituted A-ring as well as 4-methoxy substituted B-ring are an important feature underlying its tubulin binding and antiproliferative effects [46]. The presence of the hydroxyl group in the ring B has no notable role in the bioactivity of CA-4 [46], suggesting that it may be a possible region to explore for CA-4 structural modifications.

### 3.3. Noscapine

#### 3.3.1. Mechanism of Action

Noscapine is a non-narcotic natural phthalideisoquinoline alkaloid that is originally isolated from the opium poppy *Papaver somniferum* [81] (Figure 5B, Table 1D). Noscapine and its derivatives (together referred to as noscapinoids) have attracted substantial research attention due to their fewer side effects and minimal toxicity to normal tissues compared to classical chemotherapy drugs [82]. One potential explanation for the specificity of noscapine to cancer cells and its minimal toxicity to normal tissues is that normal cells may be resistant to noscapine compared to cancer cells, which divide more rapidly than normal cells and, therefore, frequently pass through a phase of vulnerability to mitotic spindle poisons. The possible resistance of normal cells to the therapeutic dose of noscapine may be potentially derived from efficient repair of noscapine-induced mitotic apparatus damage in normal cells, compared to cancer cells.

Moreover, most noscapinoids are not substrates for the Pgp drug efflux pump and several of them showed synergistic interaction with other MTAs [48,83]. Noscapine has completed phase I and II clinical trials in patients with myeloma, lymphoma, or leukemia, however, the trials were terminated due to the lack of funding (in the case of the lymphoma and leukemia trial, ClinicalTrials.gov Identifier: NCT00912899) or clinical response (in the case of the myeloma trial, ClinicalTrials.gov Identifier: NCT00183950).

#### 3.3.2. Tubulin Binding Sites

Noscapine and its derivatives bind tubulin at or near the colchicine site, and inhibit microtubule dynamics without causing gross depolymerization of microtubules [81,84].

#### 3.3.3. Structure–Activity Relationships

A number of noscapinoids with antiproliferative effects superior to the parental noscapine have been synthesized recently [43]. One notable derivative is 9–bromonoscapine (Figure 5B), which binds tubulin with greater affinity than noscapine and have activity against drug-resistant xenograft tumors without any evident toxicity [83]. Together, noscapinoids represent a unique group of MTAs that could be used as a promising lead for the development of novel anticancer agents.

### 3.4. Maytansinoids

Maytansinoids are MTAs derived from maytansine (Figure 5C). Maytansine is a benzoansamacrolide that was first extracted from the East African Shrub *Maytenus Serrata* and later from the bark of *Maytenus buchananii* [44,85] (Figure 5C, Table 1D). Maytansine exhibited remarkable cytotoxic activity against diverse cancer cell lines and inhibited tumor growth in vivo [45,85,86]. Although maytansine as single agent failed in human clinical trials, due to lack of tumor specificity and unacceptable toxicity, it was recently approved by the US Food and Drug Administration as part of an antibody-drug conjugate (ADC) for the treatment of advanced breast cancer [87].

#### 3.4.1. Mechanism of Action and the Tubulin Binding Sites

Maytanisine and maytansinoids destabilize microtubules, and earlier studies suggested that the compound occupies the vinca site on tubulin. However, recent X-ray crystallography studies demonstrated that maytansine occupies a unique site (termed maytanisine site) on β-tubulin that is different from the vinca domain [12]. The maytansine-tubulin interaction involves hydrogen bonds between the carbonyl groups at position 7a of maytansine and residues Asn102 and Lys105 of β-tubulin; hydrogen bonds between the hydroxyl/carbonyl oxygens at position 1 of maytansine and Val181 of β-tubulin; and hydrophobic interactions between the methyl groups at position 6a of the compound and residues Asn101, Asn102, Val182, Phe404, and Tyr408 of β-tubulin [12]. Notably, the unrelated microtubule destabilizing agents, rhizoxin F and PM060184 also occupy the maytansine site [12].

#### 3.4.2. Structure–Activity Relationships

Through a semi-synthesis strategy, a number of maytansine analogs (DM1, DM3, and DM4) that have disulfide or thiol groups that favor covalent linkage with monoclonal antibodies have been obtained [88,89,90]. Multiple structure–activity relationship studies on maytansine have shown that the C4–C5 epoxide moiety, the carbinolamide at C9, and double bonds at C11 and C13 are essential for optimal bioactivity. Moreover, the ester side chain (N-acyl-N-methyl-L-alanyl) at C3 is also important for bioactivity and its corresponding L-epimers are ~100-fold more active than the unnatural N-methyl-D-alanyl moiety. Notably, the side chain can be modified to generate maytansinoids bearing disulfide or thiol groups without losing bioactivity [44,85,88,90].

### 3.5. Chalcones and Quercetin

Additional plant-derived microtubule destabilizing agents that exhibited bioactivity in in vivo tumor xenograft models or that have undergone clinical trials include chalcones and quercetin [91,92]. Chalcones and quercetin are among the most important classes of flavonoids and are ubiquitously found across the plant kingdom.

#### Mechanism of Action and Tubulin Binding Sites

They occupy the colchicine site on tubulin and prevent microtubule polymerization [49,91]. Besides tubulin, these agents also target several other cellular proteins and have myriads of biological effects, including anti-oxidant and anti-inflammatory activity, in addition to their antitumor activity. Accordingly, these compounds may also have the potential for the treatment of chemotherapy-induced oral mucositis [93].

## 4. Concluding Remarks and Future Perspectives

Microtubule cytoskeleton is one of the most succesful targets in cancer therapy, and MTAs, especially paclitaxel and vinca alkaloids, play a dominant role in the treatment of diverse cancer types. However, the development of resistance by cancer cells, the reduced pharmacokinetic profile, and the severe side effects associated with their formulations often present challenges to the clinical applicability of the MTAs. Several techniques have been recently developed to avoid these issues, including nanoparticle delivery, covalent linkage to the fatty acid docosahexanoic acid, encapsulation in lipid complexes, and conjugation to antibodies. While these techniques are possibly a good approach for older generation MTAs, the future of MTAs probably lies with newer agents that more effectively evade these serious problems.

Plants are a major source of MTAs. The search for novel MTAs from plants is necessary, as it will provide a greater range of lead compounds. Notably, the production of biologically active secondary metabolites in plants are less affected by both abiotic (salinity, pollutants, light, and temperature) and biotic (space competition, predation, fouling, and presence/absence of bacterial symbionts) factors, compared to marine organisms and microorganisms [94]. Moreover, plants are characterized by the production and storage of a large number of diverse, complex mixtures of secondary metabolites [95], thus suggesting that plants are a unique source for a more consistent supply of novel, pharmacologically active MTAs.

To date, only a small portion of the world’s plant biodiversity has been exploited for the search for MTAs, thus indicating that there exists a huge opportunity for the discovery of novel MTAs from plants. Although major challenges, such as access and supply, complications in high-throughput screening due to the complexities of plant extracts, and the high cost of creating plant collections limit the interest in the search for MTAs from plants, recent advances in synthetic methodologies, fractionation and analytic methods, and genetic engineering provide hope to effectively overcome some of these challenges, including the supply issues and the screening difficulties from crude and pre-fractionated extracts. It seems that the future of plant-derived MTAs likely depend on the key features that include robust anticancer activity, low toxicity to normal tissues, and the ability to overcome resistance to existing MTAs. Identification of MTAs with these ideal features from plants would undoubtedly benefit the development of potential MTAs for cancer therapy.

As described herein, the plant-derived MTAs, taccalonolides, persin, curcumin, combretastatins, noscapine, maytansine, chalcones, and quercetin, have exhibited improved anticancer properties when compared with the taxanes and vinca alkaloids. Of note, curcumin, combretastatins, noscapine, maytansine, and quercetin have already undergone clinical trials evaluating their efficacy against various cancers [43,46,63,87,93] (Table 2). Because targeted delivery of cytotoxic agents to cancer cells increases the percentage of drug molecules that reach the tumor, and thereby lowers the minimum effective dose and increases the maximum tolerated dose, maytansine ADCs may possibly have a promising future as a clinically succesful MTA among these compounds. A complete understanding of determinants of toxicities of the maytansine ADCs and steps to increase therapeutic index are among the key areas for further improvement. To increase the therapeutic index of maytansine ADCs, improvements can be made either in the efficacy of the maytansine to reduce the minimum effective dose or in tumor specificity to enhance the maximum tolerated dose. Moreover, many maytansine ADCs are substrate for P-gp. Accordingly, more maytansine ADCs with hydrophilic linkers are needed to effectively evade P-gp-mediated drug resistance by cancer cells. Among MTAs that are in pre-clinical studies, taccalonolides are promising candidates for clinical development. Taccalonolides have shown excellent antitumor activity against xenograft models, including tumor models that are resistant to taxanes [34,50]. Although persin has shown improved anticancer properties, relative to paclitaxel, in cell-based studies [36], they have not been tested in animal models of human cancers. As such, there is still a need to establish in vivo studies with the compound, so that attempts to its translation into clinical practice can be improved. Nevertheless, the existing information on these agents provide a strong rationale for the continued exploration of their clinical potential, as well as the design and synthesis of more effective analogues or prodrugs, through the application of chemical methodologies, including total or combinatorial synthesis.

## Figures and Tables

**Table 2:** The current stage of development and the clinical trial information of taccalonolide, persin, curcumin, combretastatin, noscapine, maytansinoids, chalcones, and quercetin. The clinical trial information was obtained from the NIH ClinicalTrials.gov (https://clinicaltrials.gov/) by using the search criteria “cancer” and the “compound name” in the database. N/A indicates not applicable.

Compound	Stage of Development	Clinical Trial		
		Active (NCI clinical trial identifier, Study phase, Year of study start)	Completed (NCI clinical trial identifier, Study phase, Year of study start–study completed)	Withdrawn/Terminated/Suspended (NCI clinical trial identifier, Study phase, Year of study start–study withdrawn/terminated/suspended)
Taccalonolide	Pre-clinical (in vitro cell-based studies and in vivo human tumor xenograft studies in mice)	-	-	-
Persin	Pre-clinical (in vitro cell-based studies)	-	-	-
Curcumin	Clinical (Total 60 clinical trials: 22 active, 26 completed, and 12 withdrawn/terminated/suspended trials)	NCT04403568, Early Phase 1, 2020 NCT02724202, Early Phase 1, 2016 NCT03980509, Phase 1, 2020 NCT01859858, Phase 1, 2013 NCT01294072, Phase 1, 2011 NCT02598726, Phase 1, 2016 NCT02336087, Phase 1, 2016 NCT04294836, Phase 2, 2020 NCT00745134, Phase 2, 2020 NCT02724618, Phase 2, 2016 NCT04266275, Phase 2, 2020 NCT00745134, Phase 2, 2008 NCT03192059, Phase 2, 2017 NCT03598309, Phase 2, 2019 NCT02782949, Phase 2, 2017 NCT03493997, Phase 2, 2017 NCT03769766, Phase 3, 2019 NCT02064673, Phase 3, 2014 NCT03847623, Phase N/A, 2017 NCT03865992, Phase N/A, 2019 NCT01948661, Phase N/A, 2014 NCT03431896, Phase N/A, 2018	NCT01160302, Early Phase 1, 2010–2016 NCT01035580, Phase 1, 2010–2012 NCT01333917, Phase 1, 2010–2013 NCT00027495, Phase 1, 2001–2007 NCT01201694, Phase 1, 2011–2014 NCT01042938, Phase 2, 2008–2011 NCT02439385, Phase 2, 2015–2019 NCT03072992, Phase 2, 2017–2019 NCT01490996, Phase 1/2. 2012–2017 NCT00192842, Phase 2, 2004–2010 NCT01740323, Phase 2, 2015–2018 NCT00094445, Phase 2, 2004–2014 NCT02556632, Phase 2, 2015–2016 NCT02017353, Phase 2, 2013–2016 NCT00641147, Phase 2, 2010–2016 NCT00365209, Phase 2, 2006–2011 NCT02100423, Phase 2, 2014–2018 NCT01246973, Phase 2/3, 2011–2015 NCT01712542, Phase N/A, 2012–2013 NCT03290417, Phase N/A, 2017–2019 NCT01975363, Phase N/A, 2013–2016 NCT01917890, Phase N/A, 2011–2013 NCT03211104, Phase N/A, 2007–2015 NCT00113841, Phase N/A, 2004–2009 NCT03482401, Phase N/A, 2017–2019 NCT00927485, Phase N/A, 2007–2016	NCT01608139, Phase 1, 2012 NCT00247026, Phase 1/2, 2007 NCT02300727, Phase 1/2, 2015–2018 NCT02095717, Phase 2, 2014–2018 NCT00852332, Phase 2, 2009–2017 NCT02944578, Phase 2, 2017 NCT01269203, Phase 2, 2012 NCT00248053, Phase 2, 2005 NCT00969085, Phase 2, 2012 NCT00003365, Phase N/A, 1996–2006 NCT00118989, Phase N/A, 2005–2012 NCT00176618, Phase N/A, 2004–2007
Combretastatin	Clinical (Total 17 clinical trials: 1 active, 11 completed, and 5 withdrawn/terminated/suspended trials)	NCT02576301, Phase 1/2, 2015	NCT00395434, Phase 1, 2006–2007 NCT00960557, Phase 1, 2009–2010 NCT00003698, Phase 1, 1998–2003 NCT00003768, Phase 1, 1998–2001 NCT01240590, Phase 1/2, 2011–2016 NCT00653939, Phase 2, 2008–2011 NCT00060242, Phase 2, 2003–2008 NCT00113438, Phase 2, 2005–2007 NCT02132468, Phase 2, 2014–2016 NCT02279602, Phase 2, 2014–2016 NCT00699517, Phase 3, 2008–2013	NCT01085656, Phase 1, 2011–2016 NCT00077103, Phase 1/2, 2003–2007 NCT00507429, Phase 2/3, 2007–2011 NCT02641639, Phase 2/3, 2016–2017 NCT01701349, Phase 3, 2015–2017
Noscapine	Clinical (Total 2 clinical trials: 2 terminated trials)			NCT00912899, Phase 1, 2007–2010 NCT00183950, Phase 1/2, 2000–2006
Maytansinoids as ADC	Clinical (Total 92 clinical trials: 42 active, 37 completed, 13 withdrawn/terminated/suspended trials)	NCT04189211, Phase 1, 2017 NCT03364348, Phase 1, 2017 NCT03102320, Phase 1, 2017 NCT04042051, Phase 1, 2019 NCT03552471, Phase 1, 2018 NCT02996825, Phase 1, 2017 NCT04296942, Phase 1, 2020 NCT02390427, Phase 1, 2015 NCT03126630, Phase 1/2, 2018 NCT04298918, Phase 1/2, 2020 NCT03816358, Phase 1/2, 2019 NCT01565200, Phase 2, 2012 NCT03832361, Phase 2, 2020 NCT03418558, Phase 2, 2015 NCT02675829, Phase 2, 2016 NCT01494662, Phase 2, 2012 NCT01904903, Phase 2, 2013 NCT01853748, Phase 2, 2013 NCT04351230, Phase 2, 2020 NCT02452554, Phase 2, 2015 NCT03225937, Phase 2, 2012 NCT04419181, Phase 2, 2020 NCT03894007, Phase 2, 2019 NCT00781612, Phase 2, 2008 NCT04341181, Phase 2, 2020 NCT02314481, Phase 2, 2017 NCT04197687, Phase 2, 2020 NCT04266249, Phase 2, 2020 NCT04274426, Phase 2, 2020 NCT03587311, Phase 2, 2018 NCT02465060, Phase 2, 2015 NCT03784599, Phase 2, 2018 NCT03726879, Phase 3, 2019 NCT01966471, Phase 3, 2014 NCT04296890, Phase 3, 2020 NCT01702571, Phase 3, 2012 NCT01772472, Phase 3, 2013 NCT03084939, Phase 3, 2017 NCT03529110, Phase 3, 2018 NCT04209855, Phase 3, 2019 NCT04185649, Phase 3, 2018 NCT02226276, Phase N/A, 2015	NCT03153163, Phase 1, 2017–2018 NCT02696642, Phase 1. 2016–2019 NCT01439152, Phase 1, 2011–2019 NCT01513083, Phase 1, 2012–2014 NCT02824042, Phase 1, 2016–2019 NCT02751918, Phase 1, 2016–2019 NCT02254018, Phase 1, 2002–2014 NCT02038010, Phase 1, 2014–2017 NCT01816035, Phase 1, 2014–2017 NCT02605915, Phase 1, 2015–2019 NCT00934856, Phase 1/2, 2009–2013 NCT00875979, Phase 1/2, 2009–2011 NCT00951665, Phase 1/2, 2009–2013 NCT01638936, Phase 1/2, 2012–2018 NCT01001442, Phase 1/2, 2010–2016 NCT01470456, Phase 2, 2011–2014 NCT01472887, Phase 2, 2012–2016 NCT0261014, Phase 2, 2015–2019 NCT03023722, Phase 2, 2017–2019 NCT02924883, Phase 2, 2016–2020 NCT02289833, Phase 2, 2014–2018 NCT03106077, Phase 2, 2017–2019 NCT01975142, Phase 2, 2013–2019 NCT02254005, Phase 2, 2002–2014 NCT00679211, Phase 2, 2008–2011 NCT00509769, Phase 2, 2007–2009 NCT01196052, Phase 2, 2010–2013 NCT02999672, Phase 2, 2016–2018 NCT00679341, Phase 2, 2008–2012 NCT00943670, Phase 2, 2009–2011 NCT02420873, Phase 2, 2015–2017 NCT00829166, Phase 3, 2009–2015 NCT02631876, Phase 3, 2016–2020 NCT0112018, Phase 3, 2010–2016 NCT01419197, Phase 3, 2011–2015 NCT02131064, Phase 3, 2014–2018 NCT02658734, Phase 4, 2016–2019	NCT02221505, Phase 1, 2014–2015 NCT03045393, Phase 1, 2017–2018 NCT03455556, Phase 1, 2018–2020 NCT02947152, Phase 1, 2016–2017 NCT02318901, Phase 1/2, 2014–2018 NCT02658084, Phase 1/2, 2017–2018 NCT03836157, Phase 2, 2019 NCT02725541, Phase 2, 2016–2019 NCT02839681, Phase 2, 2016–2018 NCT01702558, Phase 2, 2012–2017 NCT01440179, Phase 2, 2011–2014 NCT01641939, Phase 2/3, 2012–2016 NCT02144012, Phase 3, 2014–2016
Chalcones	Pre-clinical (in vitro cell-based studies)	-	-	-
Quercetin	Clinical (Total 10 clinical trials: 7 active, 1 completed, and 2 withdrawn/terminated trials	NCT01912820, Phase 1, 2014 NCT03493997, Phase 2, 2017 NCT01961869, Phase 2, 2013 NCT03476330, Phase 2, 2018 NCT04252625, Phase 2, 2020 NCT02195232, Phase 2/3, 2015 NCT04267874, Phase N/A, 2019	NCT01732393, Phase 1/2, 2010–2012	NCT02989129, Early Phase 1, 2018–2020 NCT00003365, Phase N/A, 1996–2006
